# High Sensitivity Carbon Nanotubes Flow-Rate Sensors and Their Performance Improvement by Coating

**DOI:** 10.3390/s100504898

**Published:** 2010-05-14

**Authors:** Xing Yang, Zhaoying Zhou, Dingqu Wang, Xiaoli Liu

**Affiliations:** 1 MEMS Lab, Department of Precision Instruments & Mechanology, Tsinghua University, 100084, Beijing, China; E-Mails: zhouzy@tsinghua.edu.cn (Z.Z.); wangdq@tsinghua.edu.cn (D.W.); liuxiaoli09@163.com (X.L.); 2 State Key Lab of Precision Measurement Technology and Instrumentation, Tsinghua University, 100084, Beijing, China

**Keywords:** flow-rate sensor, carbon nanotube, hysterisis error, coating

## Abstract

A new type of hot-wire flow-rate sensor (HWFS) with a sensing element made of a macro-sized carbon nanotube (CNT) strand is presented in this study. An effective way to improve repeatability of the CNT flow-rate sensor by coating a layer of Al_2_O_3_ on the CNT surface is proposed. Experimental results show that due to the large surface-to-volume ratio and thin coated Al_2_O_3_ layer, the CNT flow-rate sensor has higher sensitivity and faster response than a conventional platinum (Pt) HWFS. It is also demonstrated that the covered CNT flow-rate sensor has better repeatability than its bare counterpart due to insulation from the surrounding environment. The proposed CNT flow-rate sensor shows application potential for high-sensitivity measurement of flow rate.

## 1. Introduction

Hot-wire (or hot-film) flow-rate sensors, with their wide application in fluid mechanics measurements, are predominantly used for measurement in low-velocity flow or low turbulence flow. HWFS is characterized by high spatial and time resolution, high frequency band, low background noise, simple apparatus, and low cost. With the recent developments of micro-fluid, microelectromechanical systems (MEMS), and the lab-on-a-chip, a HWFS is now required to accommodate higher sensitivity for fluid mechanics measurement in small scale. According to the principles (convective heat transfer), HWFS should have a large surface-to-volume (S/V) ratio, large temperature coefficient of resistance (TCR), and large slenderness ratio to improve their sensitivity. As a novel material, CNTs have exhibited excellent sensing properties [1], which have been utilized forgas sensor [2–4], temperature [5,6] and humidity sensors [7,8], pressure sensor [9,10], chemical sensor [11,12], biosensor [13,14], and flow sensor [15,16]. In relation to CNTs-HWFS, Victor *et al*. [15] proposed a bulk multi-walled carbon nanotube (MWCNT) element for anemometry sensors and discovered that it could be operated with very low power consumption (μW). Ghosh *et al*. [16] reported that the flow of a liquid on single-walled CNT bundles could induce a voltage that fits the logarithmic relation of velocity in the fluid. Their works showed the device’s potential for nanotubes as sensitive flow sensors. In recent years, macro-sized CNT strands (MSCNTS) composed of many individual CNTs at centimeter lengths were synthesized [17–19], which should very suit HWFS due to their large S/V ratio and large slenderness. Other advantages of MSCNTS flow sensors include high mechanical strength and low cost.

In this paper, a MSCNTS-HWFS is proposed, which demonstrates higher sensitivity and faster response than conventional Pt HWFS. To improve MSCNTS-HWFS performance, a thin oxide layer is deposited on the CNTs’ surface in the MSCNTS. The coated MSCNTS-HWFS shows better stability and repeatability than the bare counterpart.

## 2. Analysis and Design

HWFS is based on convective heat transfer from a heated wire or film element placed in a fluid flow [20–22]. Fluid velocity is measured by sensing changes in heat transfer from an electrically heated hot-wire exposed to the fluid. HWFS can be operated in different modes, wherein the two most important modes are constant current (CC) mode and constant temperature (CT) mode [21]. In constant current dissipation, the temperature of a heated hot-wire decreases with the increasing of the fluid’s flow velocity. In steady state, the heat balance equation for the HWFS is given by (1) [21,23]:

(1) $$Q_{J}=Q_{C}+Q_{\text{FC}}+Q_{R}$$

where *Q*_J_ is the generated power from Joule heating; *Q*_C_ is the sum of conductive losses involving (1) end loss of the support prongs and (2) longitudinal thermal conduction along the hot-wire; *Q*_FC_ is the heat loss due to forced convection; and *Q*_R_ is the heat loss due to the heat radiation, which can be ignored when the temperature of the hot-wire is below 300 °C.

If conductive losses and heat radiation loss are ignored, from Equation (1), Joule law, heat-transfer and hot-wire anemometer theories, the cylindrical hot-wire should have the following equation [21,23]:

(2) $$Q_{\text{FC}}=I_{w}^{2}\cdot R_{w}\approx \pi \mathrm{dlh}(U)(T_{w,\infty }-T_{f})[1-\frac{2l_{c}}{l}\text{tanh}(\frac{l}{2l_{c}})]$$

where *d* and *l* are the diameter and the length of the hot-wire, respectively; *I*_w_ and *R*_w_ are current and resistance of hot-wire; *T*_w, ∞_ is the temperature of the similar infinitely long hot-wire heated by a current *I*_w_; *T*_f_ is the temperature of the fluid; *l*_c_ is the “cold length” whose detail definition could be found in reference [21]; *h*(*U*) is the overall heat transfer coefficient which is related to the flow velocity *U* [23]:

(3) $$h(U)=A+{BU}^{n}$$

where *n* is a fitting factor that depends on hot-wire geometry; *A* accounts for the natural convection as well as the conduction end loss, and *BU^n^* is the forced convection term.

In CC-mode, the flow rate is measured by monitoring the voltage of the MSCNTS-HWFS and we have:

(4) $$V_{w}=\frac{\pi \mathrm{dl}(A+B\cdot U^{n})(T_{w,\infty }-T_{f})[1-\frac{2l_{c}}{l}\text{tanh}(\frac{l}{2l_{c}})]}{I_{w}}$$

From Equation (4), we know that under a given current, there is a definite relationship between *U* and *V*_w_. Differentiating Equation (4):

(5) $$\frac{{dV}_{w}}{dU}=\frac{\pi \mathrm{dl}(T_{w,\infty }-T_{f})[1-\frac{2l_{c}}{l}\text{tanh}(\frac{l}{2l_{c}})]}{I_{w}}\cdot n\cdot B\cdot U^{n-1}$$

Parameter *B* can be experientially expressed as [20]:

(6) $$B=\frac{0.57{\mathrm{kA}}_{s}}{d}{\left(\frac{\mu C_{p}}{k}\right)}^{0.33}{\left(\frac{\rho d}{k}\right)}^{0.5}=\frac{0.57k\cdot \pi d\cdot l}{d^{0.5}}{\left(\frac{\mu C_{p}}{k}\right)}^{0.33}{\left(\frac{\rho }{k}\right)}^{0.5}=0.57k\cdot \pi d^{0.5}\cdot l{\left(\frac{\mu C_{p}}{k}\right)}^{0.33}{\left(\frac{\rho }{k}\right)}^{0.5}$$

where *k*, *μ* and *ρ* are the thermal conductivity, the dynamic viscosity and the density of the fluid, respectively; *C*_p_ is the specific heat of the fluid at constant pressure; *A*_s_ is surface area of the wire.

From Equation (5), we know that we should maximize *dV*_w_/*dU* for obtaining the large sensitivity. Assume that the diameters and resistance of the traditional metal hot-wire and the MSCNTS hot-wire are equal. And assume that MSCNTS hot-wire is consist of many individual CNTs with diameters of *d*/*m*, the number of CNTs in the strand is about *m*^2^. From Equation (6), if the power is the same, the *B* of the MSCNT is:

(7) $$B=m^{2}\cdot 0.57k\cdot \pi {\left(\frac{d}{m}\right)}^{0.5}\cdot l{\left(\frac{\mu C_{p}}{k}\right)}^{0.33}{\left(\frac{\rho }{k}\right)}^{0.5}=m^{1.5}\cdot 0.57k\cdot \pi d^{0.5}\cdot l{\left(\frac{\mu C_{p}}{k}\right)}^{0.33}{\left(\frac{\rho }{k}\right)}^{0.5}$$

From equations (6) and (7), the *B* of the MSCNT is about *m*^1.5^ times larger than that of the metal hot-wire with the same diameter. According to the analysis, high sensitivity of the MSCNTS-HWFS is mainly due to the large S/V ratio of the macro-sized CNT strand.

## 3. Experimental Details

### 3.1. Fabrication

The centimeter-long strands of MSCNTS used in our experiments are synthesized by the catalytic chemical vapor deposition (CVD) method. The CNTs in the MSCNTS are mainly multi-walled, with diameters ranging from 5 nm to 30 nm and lengths of more than 4 millimeters.

The specific fabrication steps of the MSCNTS-HWFS include:

1. A thin MSCNTS with diameters of about tens of micrometers are pulled out from a thick as-grown MSCNTS using precise tweezers;
2. Then the MSCNTS is suspended straightly between the two 4.4 mm-spacing metal prongs;
3. A drop of conducting adhesive is dripped on each contact area and is dried under 120 °C heating conditions for 2 hours for fixing the two ends of the strand and improving the contact characteristics.

**Figure 1.**
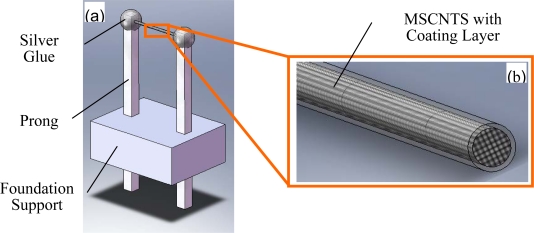
(a) Schematic diagram of the MSCNTS flow-rate sensor and (b) the enlarged view of the MSCNTS which shows the MSCNTS is composed of many individual CNTs (the MSCNTS was cut off for showing its cross-section).

Figure 1a is a schematic diagram of the MSCNTS flow-rate sensor and Figure 1b is the enlarged view of the MSCNTS which shows the MSCNTS is composed of many individual CNTs (the MSCNTS was cut off for showing its cross-section).

**Figure 2.**
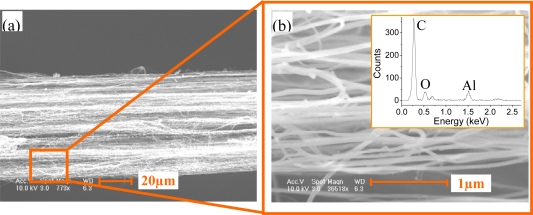
(a) MSCNTS’s SEM images wherein 50 nm Al_2_O_3_ is sputtered on the MSCNTS’s surface and (b) the enlarged view of the SEM images where inset is the EDS analysis of the MSCNTS coated with a layer of Al_2_O_3._

Aluminum oxide (Al_2_O_3_) is known to have good thermal conductivity and insulating property. To reduce the disturbance by the surrounding environment, the surface of the MSCNTS is deposited with a layer of Al_2_O_3_ via the magnetron sputtering. The thickness of the Al_2_O_3_ layer can be controlled by sputtering time. Figure 2 shows the MSCNTS’s scanning electron microscope (SEM) images where 50 nm Al_2_O_3_ is uniformly sputtered on the MSCNTS surface. Inset in Figure 2b is the EDS (Energy dispersive x-ray spectroscopy) analysis of the MSCNTS coated with a layer of Al_2_O_3_, which demonstrates that the Al_2_O_3_ has been coated on the MSCNTS’s surface.

### 3.2. Measurements

**Figure 3.**
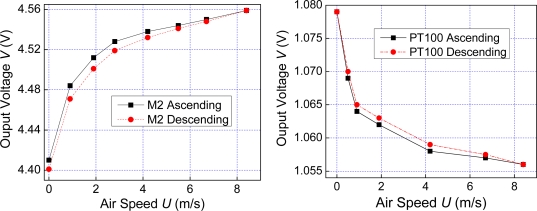
(a) Response curves of the bare MSCNTS-HWFS and (b) response curves of the Pt100.

We firstly tested the steady-state performance of the MSCNTS-HWFS in a wind tunnel where maximum air velocity was about 15 m/s, which was calibrated by a commercial HWFS to guarantee measurement accuracy. The fabricated MSCNTS-HWFS was operated in constant-current (CC) mode: a constant-current source applies a constant current on the MSCNTS, and the voltage drop of the MSCNTS-HWFS was recorded at different air velocity conditions. Although the resistance values of each MSCNTS-HWFS and Pt100 (reference commercial HWFS) were different, they retained the same power (0.01 W) in the comparable measurement. Figure 3a shows the steady-state response curves of the MSCNTS-HWFS at different air velocities wherein the CNT surface is bare.

Figure 3b shows the steady-state response curves of Pt100 at different air velocities. In CC mode, the sensitivity *S* of the HWFS can be defined as follows:

(8) $$S=\frac{V_{\text{max}}-V_{0}}{V_{0}}$$

where *V*_max_ and *V*_0_ are the output voltage at zero and maximum air velocities, respectively. According to Equation (8) and experimental curves, the bare MSCNTS-HWFS is more sensitive than the Pt100, in which the calculated sensitivities are 3.38% and 2.13%, respectively. However, the bare MSCNTS-HWFS has more hysterisis than Pt100, and the hysterisis errors are 8.23% and 4.35%, respectively.

**Figure 4.**
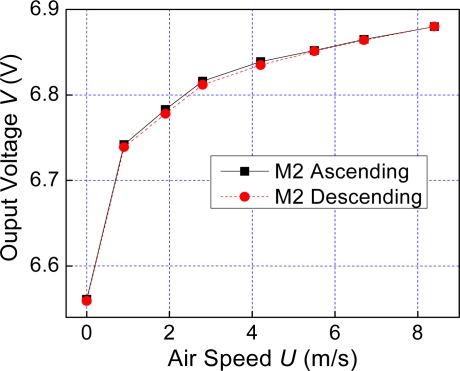
Response curves of the covered MSCNTS-HWFS.

This study also measured the response curves of the MSCNTS-HWFS coated with a layer of Al_2_O_3_. Figure 4 reveals an improvement in the curves’ repeatability at the covered MSCNTS-HWFS. The hysterisis error is reduced to 1.56%. The sensitivity of the covered MSCNTS-HWFS improves slightly (the value is 4.24%) based on the calculations.

**Figure 5.**
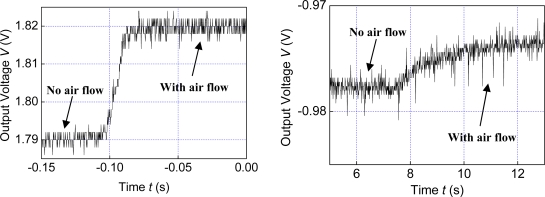
(a) Time response curve of the coated MSCNTS-HWFS and (b) time response curve of the Pt100.

We further tested the dynamic response of the coated MSCNTS-HWFS. The time responses under 8.5 m/s flow velocity were recorded as shown in Figure 5 by the withdrawing baffle plate method for producing the air flow step (both MSCNTS-HWFS and Pt100 work at a power of 0.01 W). As shown in Figure 5 the response time of MSCNTS-HWFS is about tens of microseconds, which is faster than that of the Pt100 (about several seconds).

**Figure 6.**
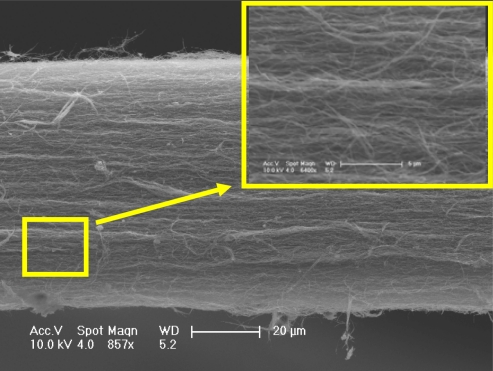
SEM images of MSCNTS (sample 2#).

We also measured the sensitivity of another MSCNTS-HWFS (sample 2#) where the MWCNTs in the strand have larger average diameter (about 50 nm) than that in the MWCNTS (sample 1#), shown in Figure 2. Figure 6 shows the SEM images of sample 2# which show that sample 2# has a looser structure than that of sample 1#. The sensitivity measurement results show that sample 2# has lower sensitivity than that of the sample 1#, which proves sample 1# has a larger S/V ratio than that of sample 2# due to the smaller average diameter of MWCNT in sample 1#. The experimental results and the SEM images also demonstrate the loose structure in the MSCNTS has better heat transfer than that of the compact structure in the MSCNTS.

The theoretical analysis and experiments prove that MSCNTS-HWFS has higher sensitivity than conventional Pt HWFS due to the large S/V ratio of the MSCNTS. It is also found that it effectively improves repeatability and reduces hysterisis error via the coating of the Al_2_O_3_ layer on the CNT surface. The reason may be that the covered CNTs are not easily disturbed by the surrounding environment and the heat could be transferred through the covered layer with high thermal conductivity. Because circumjacent humidity and gas don’t influence the resistance of the MSCNTS coated with Al_2_O_3_, the stability of the covered MSCNTS-HWFS is improved. The experimental curves also show that the MSCNTS-HWFS has higher sensitivity in low air speed range, which suits for low-speed precision measurement. The coated Al_2_O_3_ layer on the CNT surface is very thin, so MSCNTS-HWFS also has fast dynamic response.

## 4. Conclusions

We have developed a MSCNTS-HWFS characterized by higher sensitivity and faster response compared to the conventional Pt HWFS. We also propose an effective way to improve the repeatability of the MSCNTS-HWFS by coating a layer of Al_2_O_3_ on the CNT surface. Formulas and analysis are provided to explain the experimental results. MSCNTS-HWFS shows the application potential for high-sensitivity measurement of air velocity or flow rate, which can be also used for high-sensitivity measurement of fluid due to the covered insulating layer.
